# Novice Nurses' Experiences of Unpreparedness at the Beginning of the Work

**DOI:** 10.5539/gjhs.v6n1p215

**Published:** 2013-12-27

**Authors:** Mahbobeh Sajadi Hezaveh, Forough Rafii, Naiemeh Seyedfatemi

**Affiliations:** 1 School of Nursing and Midwifery, Arak University of Medical Sciences, Arak, Iran; 2 Center for Nursing Care Research, School of Nursing and Midwifery, Iran University of Medical Sciences, Tehran, Iran

**Keywords:** unpreparedness, novice nurse, transition, professional role, beginning of work, qualitative study

## Abstract

**Introduction:**

Unpreparedness of novice nurses during the process of transition to their professional role can has broad consequences for the nurse and health care system and leads to reduction of the quality of patient care. This study has been carried out with the aim of investigating the experiences of the unpreparedness of novice nurses.

**Method:**

This study was conducted qualitatively by using conventional content analysis. Participants were 21persons including 17 novice nurses, 2 supervisors, and 2 experienced nurses who were selected through purposeful sampling from four hospitals dependent on Tehran University of Medical Sciences.

**Findings:**

Participants' experiences were reflected in three main themes of "functional disability", "communicative problems", and "managerial challenges". Each of these dimensions consisted of several sub-categories. These areas had represented the inability to apply the learned knowledge in practice.

**Discussion:**

The sensitivity of health system, especially, educational mentors and nursing managers to create preparation in novice nurses by providing appropriate orientation programs at the beginning of work and the revision and amendment of nursing curriculum can solve this problem to some extent.

## 1. Background

Nursing is an independent field that its graduates are representing services as an important member of the healthcare team indifferent areas (Maben, Latter, & Macleod Clark, 2006). Nurses' knowledge and competence is based on the education and training that is taught during studying periods. Curriculum is an important subject in determining values, objectives, and educational content (Najafi Kalyani, Sharif, Jamshidi, & Karimi, 2011). The ideal training is when the related faculties grow nurses that due to increasing changes of clinical environments, new methods of caring, shorten the hospitalization period, increasing chronic diseases and technology advancement, can represent appropriate clinical services and improve the quality of patient care (Wolff, Pesut, & Regan, 2010). In fact, training competent and efficient nurses is the main target of nursing training (Bratt, 2009; Newton & McKenna, 2007; Gerrish, 2000).

At first, nursing curriculum begins with class instruction then deals with clinical training. A one of the major problems of educational system is the failure to achieve the predetermined educational goals (Najafy et al., 2011). In this regard, many studies have acknowledged that nursing graduates who are the product of nursing education system have not had enough competencies to provide high quality services at the beginning of work (Bratt, 2009; Newton & McKenna, 2007). This problem can be due to the gap between education and clinical practice.

According to Billings and Kowalski (2006) the gap between training and clinical practice (hospital) can lead to the reduction of quality patient care and also reduction of novice nurse performance quality. In nursing profession, it is believed that training and clinical practice are separate experiences (Kelly & Ahern, 2009). Even in some cases, educational educators and clinical nurses have contradictory expectations of student (Bratt, 2009; Newton & McKenna, 2007; Landers, 2000). This, in turn, increases the challenge forth nursing profession and can eventually be a threat to nursing clinical practice Billings & Kowalski, 2006). American Association of Colleges of Nursing (AACN) (2008) believes that the educational preparedness of nurses should be in such a way that they gain basic levels of competence and clinical safety (Hickey, 2010). By reviewing the texts, it was showed that clinical training efficiency of nursing undergraduate program had not been enough and nurses failed to prepare for the changing health care environments (Hickey, 2010). Hinds and Harley (2001) state that the biggest challenge for graduate students is the application of learned knowledge during studying periods in real conditions of patient care.

Many approaches such as internship programs, residency, mentorship, and perceptorship have been implemented in many countries to solve the problem of unpreparedness of newly graduated nurses (Landers, 2000). Although in many cases these programs have been extremely successful, but it still is one of the fundamental issues in nursing education that further research is needed in this area. Of course, in our country, Iran, these programs have not yet seriously carried out. And studies show that most graduates are suffering from their lack of professional preparation (Abedi, Heidari, & Salsali, 2004), following it, severe degrees of feelings and inappropriate mental and physical reactions will be evinced in them that can be effective in maintaining graduates in profession (Mooney, 2007). This problem will be presented as job dissatisfaction and will also directly affect the quality of patient care.

Although most carried studies in this case have mentioned to unpreparedness or lack of competence in new nursing graduates (Abedi et al., 2004), none of them have identified the dimensions of this problem. As the process of educating and training nurses is very expensive (Hickey, 2010), so in order to avoid wasting training resources and protecting the next generation of nursing profession, it is essential that the educational planners and scientists of this profession do some actions to grow competent and effective human workforce who are able to answer the health needs of changing community. In this regard, the identification and characterization of dimensions of this problem from the newly graduated nurses and involved staff with them can be the first step to solve this problem.

Therefore, due to the lack of research in this field in the country and the necessity to fulfill the gap in knowledge and finally to contribute to plan efficient nursing curriculum and to revise the curriculum in Iran, we aimed to do a qualitative study to determine dimensions and scope of this problem. So the current study was done with the aim of investigating unpreparedness dimensions of novice nurses.

## 2. Method

This study reports some of the findings of the doctoral thesis of researcher that has been conducted in a qualitative research method with conventional content analysis approach. 21 persons, including 17novice nurses, and 4 nursing managers participated in this study through purposeful and theoretical sampling. From new nurses, 2 persons were male and the rest were female (Table 1).

**Table 1:** Demographic characteristics of the participants

Characteristic	Mean	Range	SD
Newly graduated nurses(n=17)			
Age	24	22-25	1.5
Sex	83.33% female	-	-
	16.67% male		
Number of months of experience	6.76	3-11	4
Single/Married	8.33% married		
	91.67% Single		
Nursing managers (n=4)			
Age	43.5	39-48	4.5
Sex	75%female		
	25% male		
Number of years of experience	18.75	14-23	4.5
Single/Married	100% married	-	-

Data were collected by using deep unstructured interview and field notes. Interview was begun with this general question "talk about the first days of your job" and it was gradually focused on more specific issues. Also these explorative questions such as “Can you explain that feeling and situation with a real example to understand your feelings?”, "explain it more", and "then what happened?" were used to obtain deeper and richer data. All interviews were recorded after obtaining informed consent from participants. After doing each interview and its initial analysis, the interview was done again because of the ambiguity and the exploration of deeper data with participant. Thus, a total of 26 interviews were conducted face to face. Interview duration ranged from 25 to 120 minutes. According to Streubert and Carpenter (2007), interviews continued to data saturation. In addition, nonverbal behaviors and person's interactions with people around him/her were recording during interview as field notes in the proper position and immediately after the end of interview.

In current study, latent qualitative content analysis approach was used to analyze the data. In this method, the researcher plays the role of an interpreter, who reviewed the data for finding its meaningful parts, then codifies, classifies and organizes them. Each interview was read several times in order to immersion in the data occur. Then each word of interview was typed in word 2007 and open coding was done. At this point, the meaning units were extracted from participants' statements. Then the codes were organized by using OneNote software (Graneheim & Lundman, 2004). After several interviews, grouping was begun according to the similarity and congruence of codes. Those codes that were representing the same thing were put in a category. Then the relevant codes were extracted and put into subcategories according to their similarities. By repeating this process for each new interview, some titles were added and categories went towards perfection. Comparison and assimilation of categories belonging to a group reduced the number of initial categories. Sub-categories were grouped with events and similar occurrences as a category. Each category was named by some words that were representing its content features. The subcategories turned into the categories and lastly, the themes were determined (Polit & Beck, 2006). This process continued to form three main themes.

### 2.1 Consolidation of Study

Prolonged engagement in field and spending enough time to communicate and to gather data from September 2010 to April 2012 led to the creation of trust and rapport with the study participants and provided the possibility to gather deep data. Also, maximum variation sampling based on age, sex, place of employment, job experience, and educational status was used. To ensure that the analysis accurately reflects the experiences of the participants, member check was done during collecting and analyzing data. And, if necessary, based on the participants' suggestions, the necessary changes have been applied to the data interpretation that these actions helped to the credibility of the study.

Some parts of the raw data including interviews and products of analysis i.e. the initial codes and created categories by nursing instructors of Tehran University of Medical Sciences were investigated and audited to provide confirmability and dependability.

### 2.2 Ethical Considerations

This study was approved by the ethic committee of Tehran University of Medical Sciences. All ethical considerations such as asking permission of the hospitals where the research was done, explain the importance, objectives and methods, and especially recording the interviews, getting consent, maintaining data confidentiality at all stages and mutual decision about the time and place of the interview were observed. In addition, the researcher announced her details, phone number, and the way of achieving to the study results if the participants inclined.

## 3. Findings

Participants' experiences were reflected in three main themes of "functional disability", "communicative problems", and "managerial challenges" (Table 2).

**Table 2:** Unpreparedness categories and sub-categories of newly graduated nurses at the beginning of work

Categories	Functional disability	Communicative problems	Managerial challenges
Sub-categories	Basic and primary nursing skills	Communication with colleagues	Accountability Planning and prioritization
	Complex and specialized skills	Communication with patients	Time Management Coordination Decision making

### 3.1 Functional Disability

Participants in this study frequently mentioned to their functional defects in both accuracy and speed range. These defects included skills and primary nursing procedures such as identifying vein, adjust the serum drops, before and after surgery cares, care before diagnostic performances and also advanced and specialized nursing skills such as exchanging tracheostomy, Venous cut down, cardiopulmonary resuscitation. In this case, a novice nurse said:

"I couldn’t find the vein of persons."

Another one said:

"No matter how much I try, I still cannot find the veins well."

These defects and disabilities had been evident more in early and at the beginning of work. But sometimes in the middle of the transition period was also seen. The novice nurse has been noticed his/her unpreparedness in pre-transition period and by asking lots of questions, he/she was trying to solve the disability and to obtain his/her required skill. These defects have been in low to high dimensions and they are more due to insufficient opportunities to gain experience and to do action in training period. Also educational instructors who are incompetent to do clinical work have been proposed as one of the causes of the problem. One of the head nurses participating in the study, said:

"Of course, all things go back to the educational period. Experienced instructors are too important that cause the student to learn work during the study period. But now our most instructors cannot do clinical work that is the mentor him/herself has not done the clinical practice (has not worked in hospital), he/she does not know what the practical work is, so what things the instructor wants to teach the student. The student wants to learn training, wastes his/her time but he/she learns no practical work."

Sometimes these functional defects will be emerged in certain situations such as emergency or encountering with patients in critical situation. A novice stated about disability in doing intubation and patient resuscitation at the beginning of work (the second month):

"Patient had needed to CPR. Also, his or her vein was torn. Time was too little; I was full of stress and could not do anything."

### 3.2 Communicative Problems

One of the other aspects of defect or newly graduated nurse's deficiencies was related to communication. This communication included communication with colleagues and patients. As some novices had difficulty in connection with patient and his/her family, patients with opposite sex, patients' family, and also connection with colleagues. They were not able to communicate with other health care team members. Therefore, some health care team members were charging the novices to have a formal or severe behavior. A participant said:

"Earlier I was so shy I could not even communicate with men. I just communicated with women. I could not go into the men's' room. Earlier, guys should come to me. But I'm much better now. I can easily communicate with men. I go to their room."

Sometimes, this inability to communicate has been existed even at the level of reporting the patient status to the doctor and nurse was suffering from this problem and withstands a lot of stress. This could have serious consequences, especially for patients in emergency occasions. In this case, one of the newly graduated nurses said:

"I should call the doctor, report the patient status, it was too difficult for me, and I could not do it."

In our study, communicative problems were seen from low to high range. Some participants mentioned to lack of the ability to have a remedial communication with patient, especially in the field of answering patient. This case could reduce the patient's trust towards care team. Also, it had impact on nurse's confidence and self-esteem.

### 3.3 Managerial Challenges

One of the major problems of novice nurses had been the lack of the necessary ability to act as an administrator and coordinator of the care team in various shifts. According to the medical system that in the current status was suffering from a severe nursing shortage, sometimes, the newly graduated nurse forcefully became the shift manager and because of his/her lack of required competency, he/she involved in troubles and even sometimes he/she was ridiculed by his/her colleagues. These deficiencies had been in the areas of monitoring and controlling tasks, decision making, coordination, planning and prioritization, accountability, responsibility, time management, and delegating tasks (delegation). So that in these cases, newly graduated nurse was feeling the powerlessness, an inability to accept responsibility and lack of doing works on time. The problem would be gradually removed with doing works, getting experience, and passing time. In this case, a head nurse said:

"Newly graduated nurses, at first, are not able to plan, prioritization, and organizing ward works. Their time management is not good. They have much difficulty in doing ward managerial works."

In this case, one of the novice nurses said:

"Following works and coordinating with other parts of hospital is too difficult for me."

Another one said that:

“I felt that I couldn’t organize ward. The works were outside of my hands. I didn’t monitor my colleagues’ works, I could not control them.”

One of the factors involved in this defect is lack of the congruency of students' managerial training with determined goals for them. In most cases in managerial training, nursing students, rather than trying to be familiar with the basic principles of management that students are introduced with issues such as planning, coordination, prioritization, and surveillance and touch them in ward, are used as a surplus force in ward that all care works of other staff will be put on his/her shoulders. In this case, educational authorities should explain each trainee's objectives for head nurses and clinical authorities and they should monitor the proper implementation of trainings consistent with their purposes that student's time will not be wasted in this way.

## 4. Discussion

This study found various aspects of the unpreparedness of newly graduated nurses to work in clinical environments and showed that unpreparedness will be widely experienced in the majority of graduates at the beginning of work and causes a lot of stress for both newly graduate and care team. Findings also suggested that the lack of preparation is not limited to clinical skills but there are some defects in other skills of person especially communication and management of others. The study showed that one of the discovered themes was functional disability. Some studies consistent with our findings have showed that academic training could not equip nurses for clinical and necessary functional skills to work in clinical environment (Hickey, 2010; Gerrish, 2000). Also, some has proposed some specific causes for this problem such as educational, emotional, managerial, motivational, and organizational factors (Gerrish, 2000). However, some researchers have mentioned to the complex task of nursing as its reason and the role of clinical experience in it has been considered important for them (Abedi et al., 2004).

In this regard, Duchescher (2008) states that newly graduated nurses, after presenting in the workplace, will be quickly realized that they have not been prepared for their responsibilities and new role tasks. Also, some researchers expressed that majority of nurses experience lack of manual skills at the beginning of work. They stated that as nursing is a practice-based discipline, it is necessary to do further practical work with students about skills (Benner et al., 2010; Walrath et al., 2010). Also, a study showed that nursing training in most countries such as Sweden has had theoretical aspect and basic skills of new nursing graduates have not been satisfied (Bisholt, 2012).

Our study showed that communicative problems are being seen in a wide range in newly graduated nurses at the beginning of work. In fact, one of the important roles of nursing is remedial communication role, most of the study participants stated that, in some occasions, they were not properly prepared for this role and they were not able to communicate with a group of patients. These defects were seen in association with colleagues and doctors. This problem has been mentioned in the study of Andersson and Edberg (2010). They stated that novice nurse spend a lot of energy to communicate with others and tries to be accepted by communicating with colleagues. Also, a study showed that they are not able to communicate and respond to questions from patients' families and even to talk with doctors (Duchescher, 2008; Dyess & Sherman, 2009) and this problem causes the reduction of patients' satisfaction of care system (Scott et al., 2008).

One of researchers found that novice nurses are not able to communicate well with colleagues at the beginning of work but gradually their relationship will be improved with other nurses (Gill et al., 2010). Considering that, in nursing set in Iran, there are patients with different cultures and dialects and majority of patients are not able to make decision about their treatment and are largely dependent on nurses and medical teams and also because of the limited communication between doctor and patient, the relationship between nurse and patient will be very important, so lack of a proper communication with patient and his/her family can cause reduce the patient's consent.

Also, the sense of insecurity will be made for the patient. Walker and colleagues (2013) state that some of the personal features of novice nurses can play a role in this case. For example, open, social, and flexible persons are more comfortable to communicate with others and suffer less stress. Dachescher (2001) found that many newly graduated nurses due to the inability to establish social relationships with colleagues will be afflicted with a sense of isolation, alienation, and loneliness and consequently the marginalization experience will be created in them in workplace. In our study, managerial challenges have seen in newly graduated as unpreparedness to managerial role. Cooper and colleagues (2005) have pointed to some problems in organizing work, time management, and appearing of inappropriate reactions in the students on the verge of graduating.

Walker (2013) found that new nursing graduates are encountered with interpersonal conflicts in workplace and theDuchschery find that they lack necessary skills to manage conflict in these situations. Also, a study revealed that in the initial stages of work, novice nurses have difficulty in time management, simultaneous management of several patients, and managing critical situations. Henderson (2002) says: in twenty-first century, nursing includes a series of complex and highly specialized cares. Hence, nurses need to be armed with theoretical knowledge combined with effective practical skills to provide the needs of health care services.

Walsh and Jones (2005) say: "to pay attention to context needs about educations and to train professional effective nurses required for hospital, it should be dealt with the education based on the fitness for practice". To answer this aim, training package should be arranged in a way that leads to meet the beneficiaries' wills and needs. Daley (2001) offers the use of student-centered approaches to obtain skills, "learning how to learn" in order to raise self- leadership state, accountability, autonomy, and responsibility. The hospital structure and training method cause that enough skill does not acquire by graduates.

These newly graduated persons who are without necessary skills will encounter the structure of human resources with more failure (Dehghan Nayeri et al., 2005). Understanding the complexity of newly graduated nurses' problems may be the basis for us to move towards more inter-part cooperation in the preparation, transition, and integration of newly graduated nurses (Wolff et al, 2010; Duchescher & Cowin, 2006). Thus, considering the newly graduated nurses' problems at the beginning of work is not only a basic need to reduce their stresses but also it is an urgency to maintain retention of new nurses in the workplace (Wolff et al., 2010).

### 4.1 Limitations

Dependency of patterns and themes emerged in the context of qualitative research affects the finding applicability in different environments, however, sampling with maximum variation of different parts is one of the strengths of this study that increase its generalization to different fields. In addition, a review of studies in discussion part shows discovered implications of the common areas that is another reason for generalization and the application of findings in various fields.

### 4.2 Application of Findings

This study can help to develop a little knowledge about unpreparedness of newly graduated nurses. It can also help to integrate separate studies in this regard. And it can be a basis for generating more knowledge. Practical development based on the findings of the study helps to investigate the intensity and extent of the dimensions of the unpreparedness of newly graduated nurses. More research on this case is needed. The findings may provide a new lens for thinking about the challenges and they can apply in health policies associated with newly graduated nurses, management training and adjustment of nursing management performance. These findings can help nurse managers on effective deployment of orientation programs.

## 5. Conclusion

The findings of the study identified various aspects of unpreparedness and newly graduated nurses' challenges at the beginning of work and completed knowledge vacuum in this area. These findings can apply as a basis for future planning and reviewing nursing curriculum and tips for developing programs to provide necessary preparation in newly graduated nurses. Practical development based on the findings of the study helps to the investigation of intensity and the extent of the unpreparedness of newly graduated nurses at the beginning of work. Thus, it is necessary to apply the transition-work programs effectively. Applying these programs can reduce new graduates' stress and increase their job satisfaction and their retention. Doing more research in this area is recommended.
